# Alcohol consumption and related disorders in Iran: Results from the National Surveillance of Non-Communicable Diseases’ Survey (STEPs) 2016

**DOI:** 10.1371/journal.pgph.0000107

**Published:** 2022-11-18

**Authors:** Negar Rezaei, Naser Ahmadi, Mehran Shams Beyranvand, Milad Hasan, Kimiya Gohari, Moein Yoosefi, Shirin Djalalinia, Sahar Saeedi Moghaddam, Mitra Modirian, Forough Pazhuheian, Alireza Mahdavihezaveh, Ghobad Moradi, Farnaz Delavari, Bagher Larijani, Farshad Farzadfar

**Affiliations:** 1 Non-Communicable Diseases Research Center, Endocrinology and Metabolism Population Sciences Institute, Tehran University of Medical Sciences, Tehran, Iran; 2 Endocrinology and Metabolism Research Center, Endocrinology and Metabolism Clinical Sciences Institute, Tehran University of Medical Sciences, Tehran, Iran; 3 Department of Biostatistics, Faculty of Medical Sciences, Tarbiat Modares University, Tehran, Iran; 4 Deputy of Research and Technology, Ministry of Health and Medical Education, Tehran, Iran; 5 Deputy of Health, Ministry of Health and Medical Education, Tehran, Iran; 6 Social Determinants of Health Research Center, Kurdistan University of Medical Sciences, Sanandaj, Iran; University of Oxford, UNITED KINGDOM

## Abstract

**Background:**

Alcohol consumption is a public health concern which is illegal in Iran. Moreover, due to cultural and religious beliefs, the available population-based research findings on alcohol consumption are inadequate. We aimed to provide an estimate on alcohol consumption using a large-scale population-based survey in Iran.

**Materials and methods:**

The National Surveillance of Non-Communicable Risk Factors in Iran was a population-based survey conducted in 2016. The epidemiologic distribution of alcohol consumption and its related disorders were assessed using weighted survey methods and multiple logistic regression models. Age standardized rates were calculated using Iran’s national population census in 2016.

**Results:**

At the national level, the prevalence rates of lifetime and current alcohol consumption were 8.00% (95% CI: 7.67–8.32) and 4.04% (95% CI: 3.81–4.27), respectively. The highest prevalence was reported among 25 to 34 year-olds. Individuals of higher socioeconomic status consumed significantly greater levels of alcohol. At provincial level, the highest and lowest percentages of the current alcohol drinking rates in Iran’s provinces were, 23.92% (95% CI: 17.56–30.28) and 0.4% (95% CI: 0–1.18) in males, 1.58% (95% CI: 0.22–2.94) and 0% in females, respectively. In urban regions, the highest alcohol consumption rate was more than 22 times greater than the lowest alcohol consumption rate. Current alcohol drinkers were 2 times more prone to injury as compared to nondrinkers (OR_adj_: 2.0, 95%CI: 1.7, 2.3).

**Conclusion:**

In Iran, the prevalence of alcohol consumption is low, although there is a considerable variation of alcohol consumption at provincial level as well as in different gender groups. Therefore, preventive WHO—recommended measures should be adopted more seriously by vulnerable groups.

## Introduction

Globally, alcohol use disorder caused 2.1 deaths per 100,000 persons in 2019 [1]. The death rate was 5.44 per 100,000 in the WHO European Region and 0.61 per 100,000 in the WHO Eastern Mediterranean Region (EMR) [1]. The total alcohol per capita consumption in the population aged over 15 years demonstrates an increasing pattern from 5.5 liters of pure alcohol (ethanol) in 2005 to 6.4 liters in 2016 [2, 3]. This pattern was steady in the EMR, and showed a decline in the European Region [2, 3]. The mortality rate due to alcohol disorder in Iran was estimated at 0.28 per 100,000 in 2019 [1]. The total alcohol per capita consumption was reported to be 0.1 liters [4].

Alcohol is associated with many disorders, including dependency, alcohol-related deaths, mental and physical disorders, cardiovascular disorders, liver disease, violent and anti-social behaviors in adolescents and young people, and traffic injuries [2, 5–7]. A total of 389,100 cases of cancers were related to alcohol worldwide, representing 3.6% of all cancers (5.2% in men and 1.7% in women) [8]. Alcohol consumption is a risk factor among young people, causing disability or even death [9, 10].

Civil laws regarding alcohol trade and use in Islamic countries are considerably different, as alcohol consumption is forbidden in Islam. In Iran, the prevalence of alcohol consumption was reported at 352.4 per 100,000 persons in 2019 [1]. Hence, alcoholic beverages are either homemade or distributed through the black market [11, 12]. This form of alcohol consumption leads to various disorders such as methanol toxicity and at times even death [6, 12, 13]. Furthermore, the prevalence of alcohol consumption is underreported due to stigmas and prohibition rules [6, 11]. Therefore, reports on the prevalence of alcohol consumption are limited [14, 15] and there is no comprehensive population-based study to evaluate the provincial prevalence of alcohol consumption and its association with relevant disorders. Thus, we aimed to estimate the overall prevalence of alcohol consumption by age, gender, and province and its association with stroke, cardiac disorders, fatty liver disease, traffic injuries, and a healthy diet using a population-based survey.

## Materials and methods

### Overview

The National Surveillance of Non-Communicable Risk Factors in Iran was conducted on 31,050 people over the age of 18 years. It was a cross-sectional population-based household survey of non-communicable diseases (NCD) risk factors by sex and age carried out at the provincial level in 2016. The survey was conducted with the objective of continuously collecting information in three phases: data collection of NCD related risk factors using a questionnaire, gathering the required information through physical examinations and performing laboratory measurements. Determination of non-communicable risk factors including alcohol consumption was one of this survey’s first goals [16]. After collecting data on age, sex, and different demographics from all family members, we included family members who were 18 years or older. We also excluded people with serious physical and mental illnesses who could not be interviewed. We filled out a questionnaire, measured anthropometric indices, and collected blood and urine samples of all the participants for laboratory controls (laboratory controls were performed for individuals 25 years and older).

### Sampling

A cluster random sampling frame was considered for proportional to size sampling. For our sampling frame, we used the countrywide postal code database, which incorporates the addresses of all residential homes inside the country. Through a scientific choice of samples proportional to size, we chose samples proportional to the dimensions of rural and urban regions inside every province. A total of 3105 clusters and 31,050 Iranian adults aged 18 ≤ were selected. We used the province of Ilam–with the lowest population in Iran–to calculate the minimum sample at 95% confidence interval. 384 samples were considered as the baseline of our calculations. Sample sizes of other provinces were estimated based on the population ratios of each province to Ilam province. To account for the effect of sampling design and to control non–response error, 10% was added to the estimated sample size of each province. To reduce costs and increase productivity, for provinces with 800 or more samples calculated through weighting, it was decided that half the calculated sample size be considered along with double the weight of estimates. We considered these sampling weights in the analysis of results using survey analysis in STATA. This Survey was carried out following WHO’s recommendation to control NCDs. Additional detailed information on sample size calculation, questionnaire validation and study implementation can be found elsewhere [16]. Eventually, 31,050 participants were included in the study, and 30,541 accept to complete the questionnaires. Overall, 29,068 participants completed all items in the alcohol section’s questionnaire. Therefore, the response rate, which is calculated by dividing the number of participants who answered all the questions of the alcohol section by the total number of individuals included in the study, was approximately 93.6%.

### Definition of variables

The tool used to evaluate alcohol consumption in this study was based on WHO’s guidelines [17]. The questionnaire was first completed by interviewers who had been trained in research centers and later assigned to the fields. It included questions about lifetime and current alcohol consumption (frequency of alcohol consumed in the past year), binge drinking (defined as frequency of 6 standard drinks or more in one episode), and frequent heavy episodic drinking (defined as frequency of monthly or more episodes of binge drinking during the past year). The question on current alcohol consumption was “Did you drink alcohol in the past 12 months? Yes/ No”. The question on lifetime consumption was “Did you ever drink alcohol in your life?”. Driving under the influence (DUI) of alcohol was asked by the question “Have you ever driven a car while you were drunk? Yes/No”. A ‘standard drink’ is a measure of alcohol consumption representing a fixed amount of pure alcohol, used for future recommendations on alcohol consumption and its associated health risks. This definition varies in different countries [11]. In Iran, the amount is 10 grams, similar to Australia, and is lower than the United States and Japan [11]. As alcohol consumption is banned due to religion and law in Iran, we defined a standard drink for the participants by providing them an image of the amount of a standard drink for their better understanding. This was emphasized during the interviewers’ training sessions.

Cardiovascular disorders were defined as a patient’s response to the question “Have you been diagnosed with a cardiac disorder or stroke in the past 12 months?” Fatty liver disease was defined by an ALT level higher than 40 unites per liter. Injuries were defined as a participant’s response to the question “Did you sustain any injury in the past 12 months?” A healthy diet was defined as two servings of fish and 5 servings of vegetables or fruits per week. The wealth index (SES), which was derived from the household assets survey was analyzed using principal component analysis (PCA) and categorized into five quintiles. Physical activity was assessed by the IPAC questionnaire [18] and defined as a MET less than 6 hours.

### Ethical considerations

The Ethical Committee of the National Institute for Medical Research Development (NIMAD) approved this study under the registration code ID: IR.NIMAD.REC.1394.032. Participation in the study was voluntary. The objectives of the study were explained to all eligible individuals and written informed consent was thereafter obtained.

### Statistical analysis

First, we used weighted survey statistical methods for the descriptive reports, and prevalence. Second, age-standardization of prevalence rates was performed using the 2016 National Population Census [19] data obtained from Iran’s Statistical Center. These age-standardized measures were provided in maps. Third, the models were built distinctly to evaluate the association between alcohol consumption and the defined variables using univariate and multiple logistic regression analysis. The outcomes were cardiovascular disorders [16], suspected fatty liver disease [20], injuries [16], and healthy diet [17, 21]; the exposure was current alcohol consumption.

Univariate analysis was performed to calculate crude Odds Ratio (ORc), and 95% Confidence Interval (CI) for each pair of exposure and outcome. Wealth index (SES) [16], sex, age, smoking and physical activity [7, 22–24] were confounding variables. They were tested separately for their associations with outcomes and exposure, and all met the criteria for being confounders, so they were adjusted in the final multiple regression model to control for confounding bias. The multiple logistic regression model was used to calculate the adjusted odds ratio (ORadj), and 95% CIs applying the Enter method.

All the analyses were performed with STATA software version 14.0 (Stata Corp, College Station, TX, USA). The R statistical software version 3.4.2 (R Foundation for Statistical Computing, Vienna, Austria) was used for the plots.

## Results

The participants’ mean age was 44.4 years (range: 18 to 100 years). The majority of the participants were female (52.08%), non-smokers (85.37%), physically inactive (56.36%), and urban residents (71.09%). Table 1 shows the characteristics of the study participants.

**Table 1:** The alcohol drinking status and demographic characteristics in Iran, 2016 (National Level).

		Alcohol Drinking Status			
		Lifetime alcohol drinking (n = 29875)		Current alcohol drinking (n = 29869)	
Variables	Categories	Prevalence (95%CI)	P-value*	Prevalence (95%CI)	P-value*
Sex	Male	15.3 (14.6,15.9)	<0.001	7.6 (7.2,8.1)	<0.001
	Female	1.3 (1.2,1.5)		0.8 (0.6,0.9)	
Age	18–24 years	8.8 (7.7,9.9)	<0.001	6.6 (5.7,7.6)	<0.001
	25–34 years	11.5 (10.7,12.2)		6.9 (6.3,7.6)	
	35–44 years	9.3 (8.5,10.0)		4.5 (4.0,5.0)	
	45–54 years	6.6 (6.0,7.2)		2.5 (2.0,2.9)	
	55–64 years	4.9 (4.2,5.5)		1.7 (1.3,2.1)	
	65–70 years	4.8 (3.7,6.0)		1.1 (0.5,1.6)	
	>70	4.1 (3.3,4.9)		0.6 (0.2,1.0)	
Wealth index	Poor	5.4 (4.8,6.0)	<0.001	2.6 (2.2,3.1)	<0.001
	Second quintile	7.4 (6.7,8.1)		3.4 (2.9,3.9)	
	Third quintile	8.1 (7.3,8.9)		3.9 (3.3,4.4)	
	Forth quintile	9.4 (8.6,10.2)		4.8 (4.2,5.4)	
	Rich	10.1 (9.3,11.0)		5.6 (5.0,6.2)	
Smoking	Yes	26.6 (25.3,28.0)	<0.001	12.3 (11.3,13.3)	<0.001
	No	4.8 (4.5,5.1)		2.6 (2.4,2.8)	
Low physical activity	Yes	5.7 (5.3,6.1)	<0.001	2.8 (2.5,3.0)	<0.001
	No	9.2 (8.6,9.7)		7.6 (6.6,8.6)	
Residential status	Rural	7.1 (6.5,7.6)	<0.001	3.4 (3.0,3.8)	<0.001
	Urban	8.4 (8.0,8.8)		4.3 (4,4.6)	

* The p values are derived from chi2 test.

**Fig 1:**
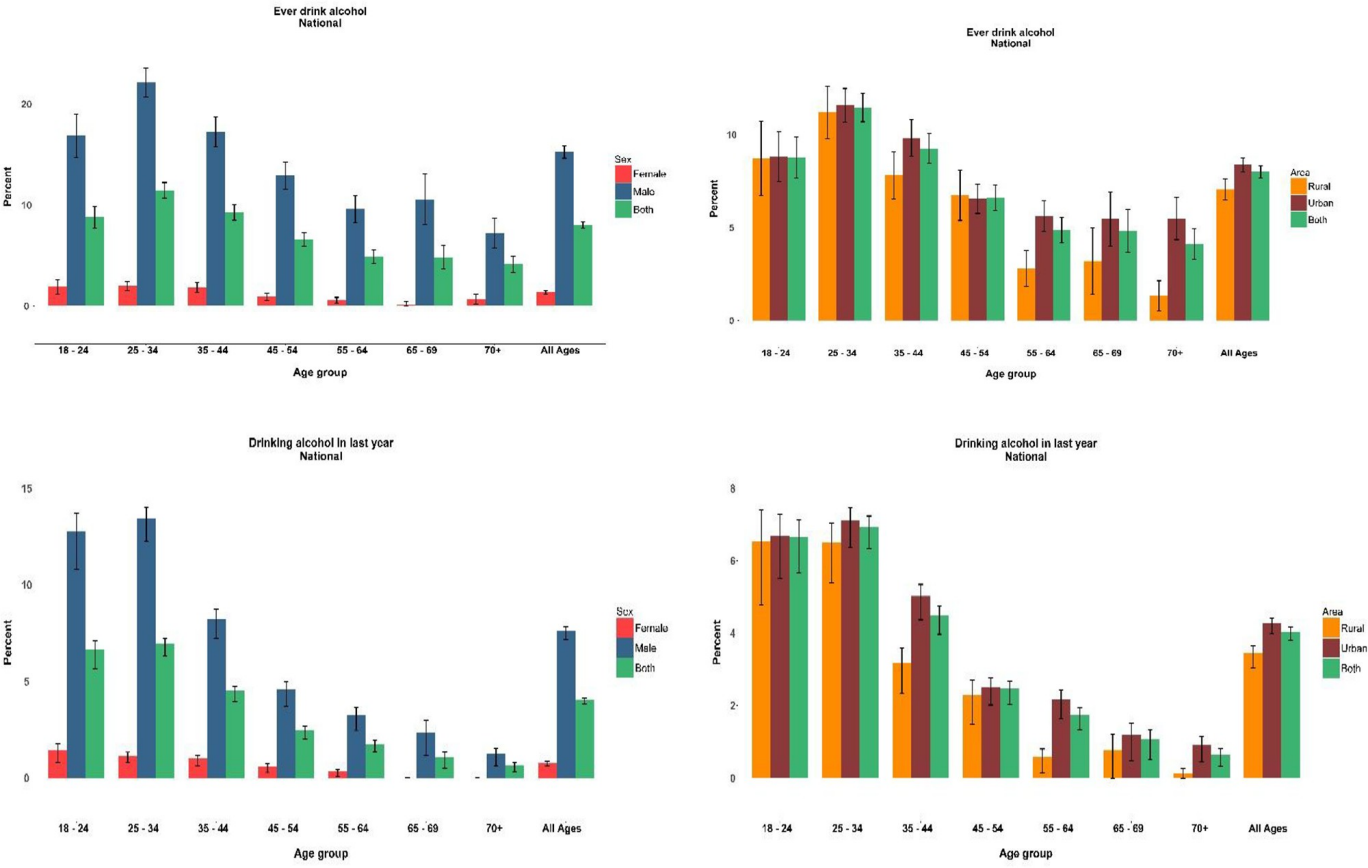
Distribution of alcohol drinking among age group by sex and residential status.

**Fig 2:**
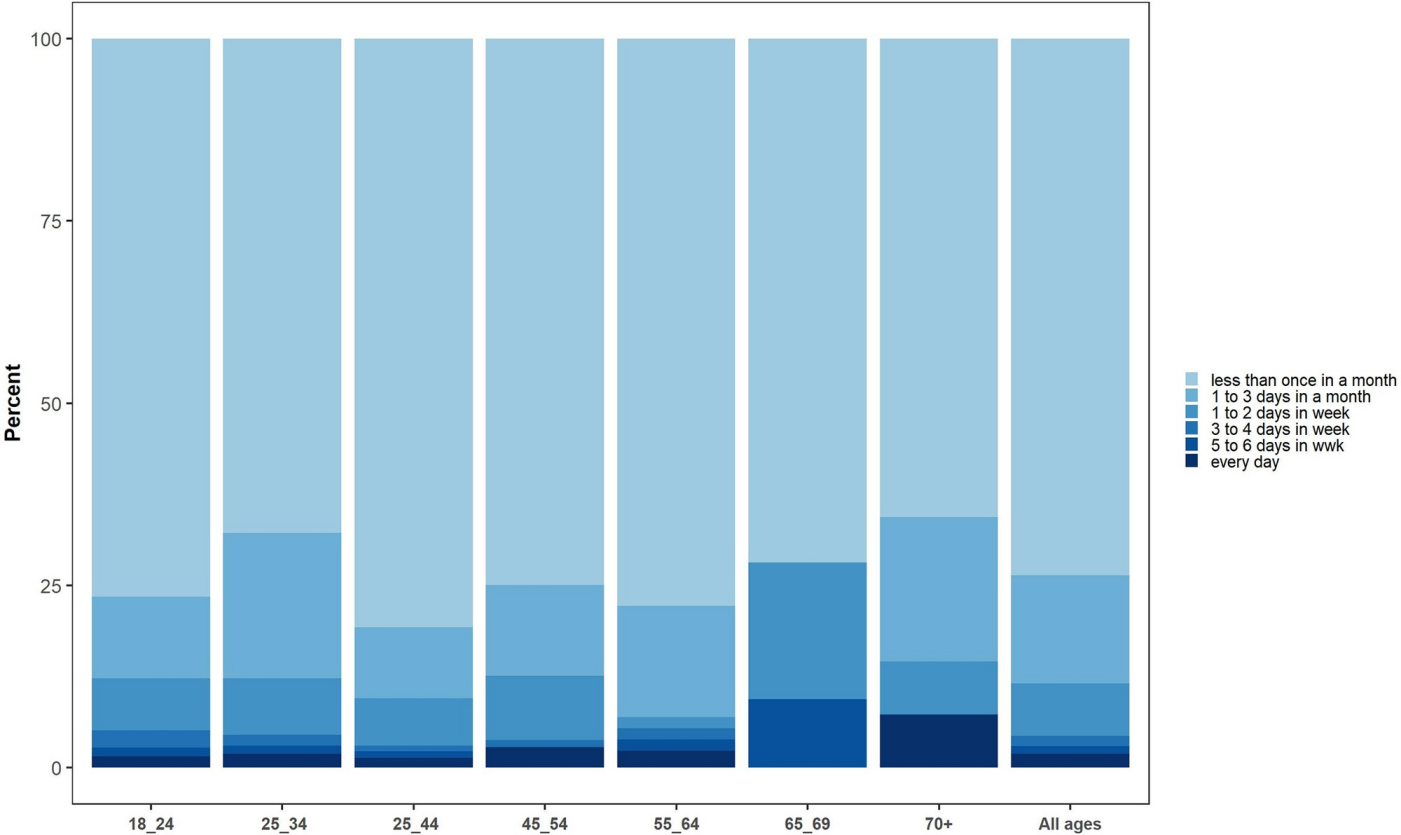
Drinking frequency in last 12 months (at national level by age groups).

At the national level, 8.00% (95% CI: 7.67–8.32) and 4.04% (95% CI: 3.8–4.3) of the participants declared lifetime and current alcohol consumption, respectively. The highest prevalence of lifetime and current alcohol consumption was reported among 25 to 34 year-olds. The prevalence of both types of alcohol consumption reduced significantly with an increase in age (p value for trend <0.001) (Fig 1). In our study, socioeconomic status (wealth index) and prevalence of current alcohol consumption were directly proportional. Persons of high SES consumed nearly twice as much alcohol compared to persons of low SES (Table 1). The response rate to frequency of alcohol use in the past year was 89.73% (1040/1159). Among respondent participants, 73.36% (763/1040) drank less than 1 day per month, 14.90% (155/1040) drank 1 to 2 days per month and only 0.20% (21/1040) drank every day (Fig 2). Regarding lifetime and current alcohol consumption, the difference in prevalence rates between males and females was significant (15.27% and 7.61% in males, vs 1.35% and 0.78% in females, respectively). Among the (predominantly male) respondents who lived in urban regions with a mean (SE) age of 35.78 (2.81) years, approximately 0.1% binge drank every day. All in all, 1.5% of participants binge drank less than 12 times a year and less than 1% practiced frequent heavy episodic drinking. However the prevalence of binge drinking among current alcohol consumers in daily, weekly, monthly, and less than 12 months a year, are 1.60%, 6.49, 15.14, and 35.03%, respectively (Table 2).

**Table 2:** Pattern of alcohol consumption in Iran in 2016 (National Level).

	Total Prevalence (95%CI)	Male Prevalence (95%CI)	Female Prevalence (95%CI)	Urban Prevalence (95%CI)	Rural Prevalence (95%CI)
Lifetime alcohol consumption					
Yes	8.00 (7.67,8.32)	15.27 (14.65,15.89)	1.35 (1.16,1.53)	8.37 (7.98,8.77)	7.07 (6.52,7.63)
Current alcohol consumption					
Yes	4.04 (3.81,4.27)	7.61 (7.16,8.06)	0.78 (0.64,0.93)	4.29 (4,4.57)	3.44 (3.04,3.85)
Binge drinking among current alcohol consumption					
Every day	1.60 (0.85,2.34)	1.74 (0.91,2.57)	0.42 (0.01,1.25)	1.64 (0.78,2.51)	1.45 (0.04,2.95)
Weekly	6.49 (5.01,7.98)	6.57 (4.99,8.15)	5.92 (1.66,10.17)	6.44 (4.75,8.13)	6.66 (3.6,9.73)
Monthly	15.14 (13.02,17.27)	15.57 (13.29,17.85)	11.7 (5.94,17.46)	15.19 (12.73,17.65)	14.99 (10.76,19.21)
Less than 12 times per year	35.03 (32.18,37.88)	35.96 (32.92,39)	27.56 (19.67,35.46)	36.07 (32.76,39.38)	31.95 (26.37,37.52)
Never	41.74 (38.8,44.68)	40.16 (37.06,43.27)	54.4 (45.55,63.25)	44.95 (39.02,50.87)	40.65 (37.27,44.04)
Past month alcohol consumption					
Yes	2.08 (1.91,2.25)	3.96 (3.63,4.3)	0.37 (0.27,0.46)	2.23 (2.02,2.44)	1.73 (1.44,2.02)
Driving under the influence of lifetime alcohol					
Yes	0.53 (0.45,0.62)	1.05 (0.87,1.22)	0.06 (0.02,0.11)	0.58 (0.47,0.68)	0.43 (0.28,0.58)

**Fig 3:**
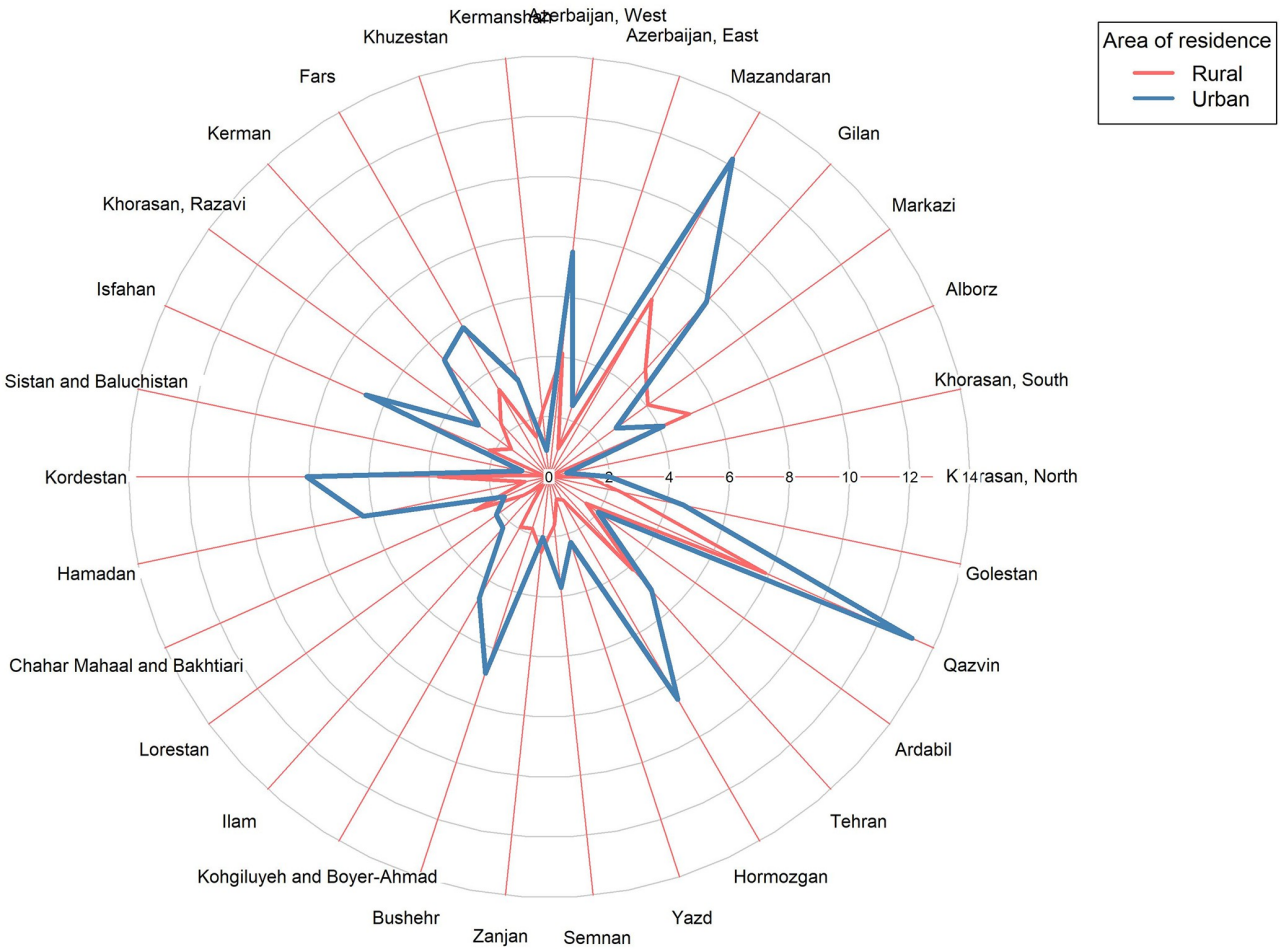
Provincial distribution of alcohol drinking (%) within the past year.

**Fig 4:**
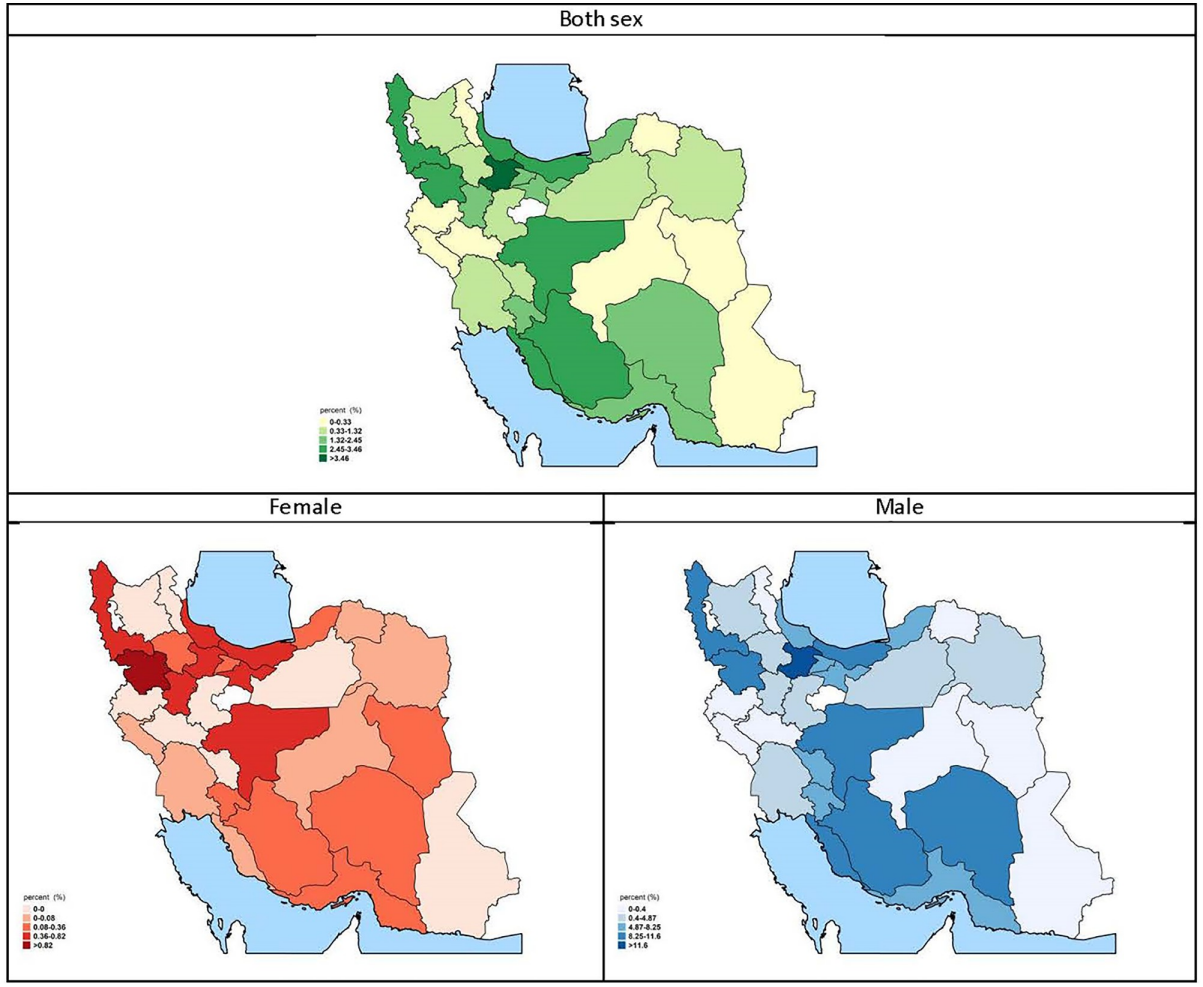
Current alcohol drinking prevalence map of Iran, 2016, https://www.openstreetmap.org/copyright.

At the provincial level, the highest and lowest percentages of age-standardized current alcohol consumption were, respectively, 13.22% (95% CI: 7.66–18.77) and 0.58% (95% CI: 0–1.51) in urban regions and 7.88% (95% CI: 3.98–11.77) and 0% in rural regions. In other words, in urban regions, the highest current alcohol consumption rate was 22 times greater than the lowest current alcohol consumption rate (Fig 3). At the provincial level, the highest and lowest rates of age-standardized current alcohol consumption were, respectively, 23.92% (95% CI: 17.56–30.28) and 0.4% (95% CI: 0–1.18) in males, 1.58% (95% CI: 0.22–2.94) and 0% in females, and 12.49% (95% CI: 9.23–15.75) and 0.46% (95% CI: 0–1.09), in both genders (Fig 4).

In Tehran (the capital of Iran), the prevalence rates of current alcohol consumption for all ages of both genders, males, and females were, 4.27% (95 CI: 3.69–4.85), 7.39% (95 CI: 6.31–8.46), and 1.32% (95 CI: 0.87–1.78), respectively.

**Fig 5:**
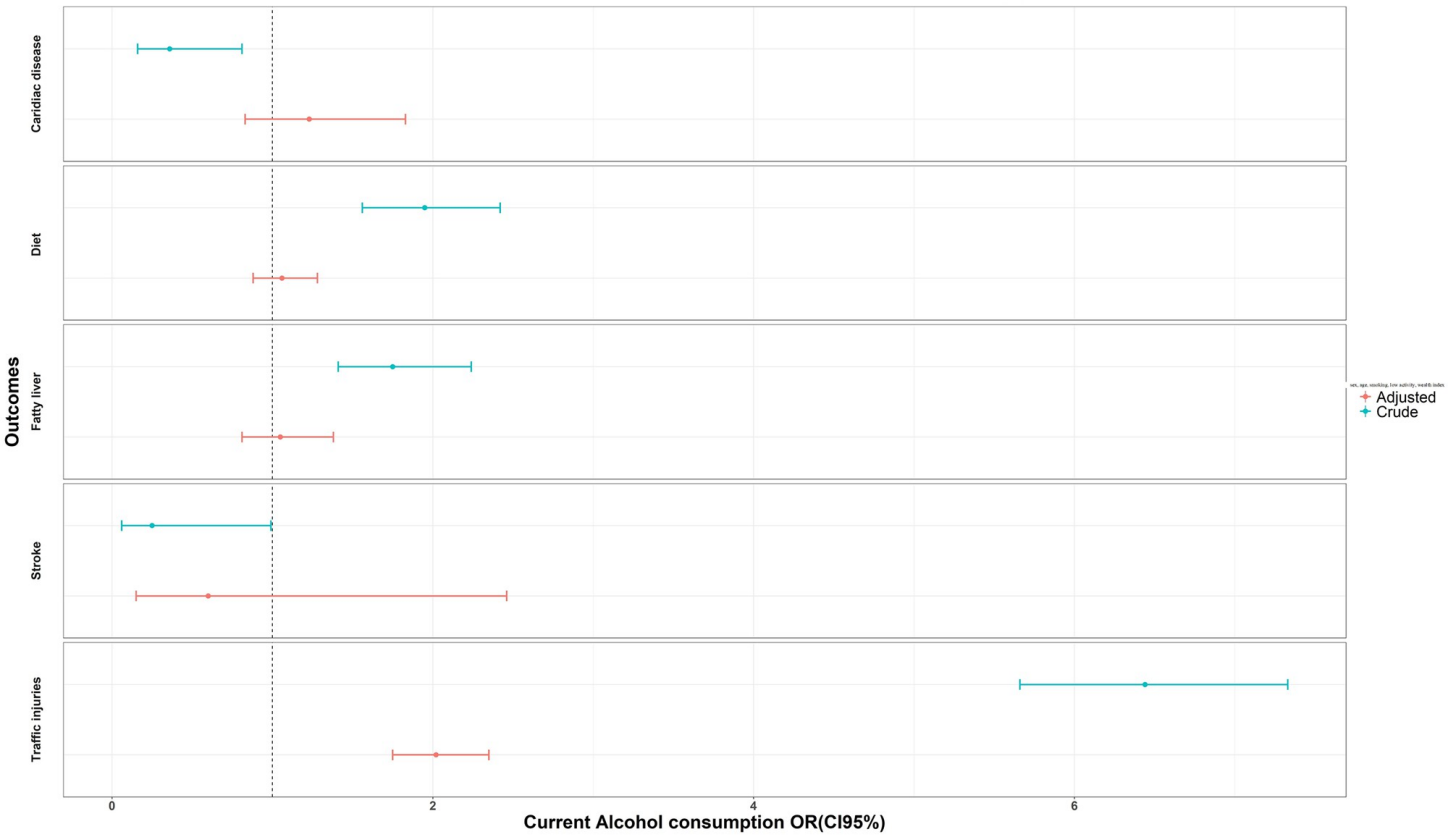
Association of alcohol related disorders with alcohol consumption in logistic regression models: Result from the National Surveillance of Non-Communicable Disease Survey (STEPs).

Results of the crude model (OR_C_) showed that compared to nondrinkers, current alcohol consumption respondents had lower risks of cardiac disorders and stroke by 70% and 80%, respectively (OR_c_ cardiac disorder: 0.3, 95% CI: 0.2, 0.8; OR_c_ stroke: 0.2, 95% CI: 0.7, 0.9). However, these reports were not significant when adjusting the model (OR_adj_) for sex, age, smoking, wealth index and physical activity confounders. Current alcohol consumption was associated with cardiac diseases but was not significant (OR_adj_ cardiac disorder: 1.2, 95%CI: 0.8, 1.8, OR_adj_ stroke: 0.6, 95% CI: 0.1, 2.4). Current alcohol drinkers were more likely to have poor dietary habits and fatty liver disease as compared to nondrinkers (OR_c_ bad dietary intake: 1.9, 95% CI: 1.6, 2.4, OR_c_ fatty liver: 1.8, 95% CI: 1.4, 2.2), but this association was not significant (OR_adj_) when controlling the aforementioned confounding variables (OR_adj_ bad dietary intake: 1, 95% CI: 0.8, 1.2, OR_adj_ fatty liver: 1, 95% CI: 0.8, 1.3). Alcohol drinkers were more prone to traffic injuries. Current alcohol drinkers sustained traffic injuries 6 times more than nondrinkers (OR_c_ injury: 6.4, 95%CI: 5.7, 7.3). The odds ratio reduced by half when adjusting for confounders and was statistically significant (OR_adj_ injury: 2.0, 95%CI: 1.7, 2.3) (Table 3, Fig 5).

**Table 3:** Association of alcohol related disorders with alcohol consumption in logistic regression models: Results from the National Surveillance of Non-Communicable Diseases Survey (STEPs).

	No Alcohol Consumption in the Past 12 Months	OR	CI 95%	p-value
Cardiac disease				
Model 1	Reference	0.36	(0.16, 0.81)	0.01
Model 2	Reference	1.23	(0.83, 1.83)	0.30
Stroke				
Model 1	Reference	0.25	(0.06, 0.99)	0.04
Model 2	Reference	0.60	(0.15, 2.46)	0.48
Diet*				
Model 1	Reference	1.95	(1.56, 2.42)	<0.001
Model 2	Reference	1.06	(0.88, 1.28)	0.54
Fatty liver disease				
Model 1	Reference	1.78	(1.41, 2.24)	<0.001
Model 2	Reference	1.05	(0.81, 1.38)	0.67
Traffic Injuries				
Model 1	Reference	6.44	(5.66, 7.33)	<0.001
Model 2	Reference	2.02	(1.75, 2.35)	<0.001

Model 1: Crude association

Model 2: Adjusted for sex, age, smoking, low activity, wealth index

*Healthy diet defined as two times having fish and 5 times having vegetables or fruit in their meal, per week

## Discussion

The prevalence of lifetime and current alcohol consumption was 8.00% (95% CI: 7.67–8.32) and 4.04% (95% CI: 3.8–4.3), respectively. The highest prevalence was reported among 25 to 34-year-old individuals. At the provincial level, the differences between the highest and lowest percentages of current alcohol consumption in males and females, and between urban and rural regions were nearly 23, 12, 12, and 7 percent, respectively. Moreover, current alcohol consumption respondents were susceptible to injury twice as much as nondrinkers.

Most studies conducted in Iran have discretely examined urban or lifetime prevalence, whereas this study has focused on urban, rural, male, female, age-standardized and all-age prevalence rates of current alcohol consumption [14, 15, 25, 26]. For instance, in the capitals of five provinces the prevalence of lifetime alcohol use was reported at a rate of 28% [15, 25]. Another population based study reported the frequency of alcohol use among the general population aged over 15 at about 2.31%. Males had almost 8 times higher prevalence of lifetime alcohol consumption compared to females (4.13% versus 0.56%) [14]. The huge difference observed in current alcohol consumption between provinces–especially among females and rural regions- may be explained by the diversion in cultural and religious beliefs among different genders and age groups, and is consistent with other studies conducted in Iran [25, 26]. Alcohol consumption among men and women varies noticeably in different countries, from nearly 10 to 20 percent to approximately 70 to 90 percent [27–29]. These rates are even lower in Islamic countries compared to other countries across the globe [30]. In Iran, women are more likely to be socially active and less exposed to people with high-risk behaviors such as narcotic drug abuse or alcohol misuse [6, 27, 31]. One report suggests an inverse relationship between socioeconomic status and alcohol consumption [31]. Nonetheless, other reports show a direct association [32], which is consistent with this study’s results. Age is directly associated with alcohol consumption. Reports show that 18 to 24-year-olds have the highest alcohol consumption rate, and the rate decreases with increasing age [33]. This study showed that alcohol consumption increased among 25 to 34-year-olds and then decreased with age, which is consistent with other reports from Iran [10, 26].

For many years, research studies indicated that light alcohol consumption had benefits for coronary diseases and stroke [34, 35]. The ‘J-shaped’ dose response of alcohol consumption and all-cause mortality, stroke, and heart disease have been misused by alcohol industries [35–39]. At the time of this study, alcohol-attributable cancer mortality rates were estimated at 5.8% [40]. However, several studies have reported that, irrespective of the dose consumed, alcohol has no protective effect on all-cause mortality and cancer incidence [37, 38, 41]. In this study, the protective effect of alcohol was seen in the univariate model with cardiac disease and stroke. Nevertheless, after adjusting for confounders, the association was no longer significant, which is consistent with the new systematic reviews available on this topic [38]. A meta-analysis did not find any relationship between alcohol consumption and reduced heart disease mortality, which implies that alcohol does not enhance health [37, 39]. However, this study is a cross-sectional study prone to unmeasured confounders that could not be adjusted for their associations and future studies are warranted on this topic. Reports indicate alcohol consumption association with fatty liver disease [40, 42]. However, in this study, we could not find any association. Worldwide, 32% of alcohol-attributable deaths result from accidental injuries, whereas 13.7% result from intentional injuries [3]. This situation is even worse and alarming in low-income countries with an increasing rate of alcohol consumption and underreporting bias in policymaking reports [3, 43, 44]. Based on our findings, the risk of injury increased more than two times in alcohol drinkers when compared to nondrinkers, which is consistent with earlier studies [43, 44].

With regards to the limitations of this study, one is that its methodological design cannot assesse causality. Although we could capture many sociodemographic confounders, there are still residual confounding which needs more detail on drugs and medication, addiction to alcohol, alcohol consumption withdraw, exact amount of alcohol consumption. We could not capture these variables due to social and legal and these information are beyond the aims of the STEPs survey [15]. However, it does illustrate a picture of the current situation of alcohol consumption prevalence in Iran. We recommend conducting cohort studies to assess the causality with assessing potential confounders during follow ups. Nevertheless, the strength of this study is its well-designed, population-weighted, cross-sectional, provincial level survey from 30 provinces of Iran. Hence, it could represent close-to-reality estimates of the existing conditions and associations between alcohol consumption and related disorders in Iran. To our knowledge, this is the first study to assess these associations to this extent, adjusting for confounders such as sex, age, wealth index and physical activity. Another limitation is the stigma of alcohol consumption in Iran due to religious and legal bans. In this study, we are not sure whether the negative responses to alcohol consumption were accurate, due to the cultural, social, legal and religious stigmas attached to it in Iran. So we estimate an under-reporting bias in the results. This selection bias could be due to the under-representation of heavy and problem drinkers, and those with severely poor health in health survey. We recommend conducting future studies using specific questionnaires and applying methodological and statistical methods [45, 46] in order to detect under-estimation and non-response bias on alcohol consumption in Iran.

## Conclusion

Although, the prevalence of alcohol consumption in Iran is lower than in developed countries, there is a considerable variation of alcohol consumption at provincial level as well as different gender groups. Also there is an association with injuries. In this regard, preventive social measures should be adapted more seriously [25, 47]. Given WHO’s global strategy [48] to reduce the harm of alcohol consumption (of the ten components of national action regarding this issue), policy makers and stakeholders in Iran could focus on awareness, health services’ response, community action, reducing alcohol intoxication, reducing the public health impact of illicit, informally produced alcohol, and monitoring & surveillance. Social, individual and public empowerment is recommended through educational programs and fighting stigmas attached to alcohol consumption. This will be accomplished through public health advocacy. Protective strategies must be considered to reduce the harm of alcohol consumption among high risk groups.
